# Mining Employees Safety and the Application of Information Technology in Coal Mining: Review

**DOI:** 10.3389/fpubh.2021.709987

**Published:** 2021-08-18

**Authors:** Li Yang, Getnet Engeda Birhane, Junqi Zhu, Jichao Geng

**Affiliations:** Department of Economics and Management, Anhui University of Science and Technology, Huainan, China

**Keywords:** coal mining safety, mining injuries, coal mining accidents, human error, human behavior, intelligent mining, internet of things, mining environmental impact

## Abstract

**Background:** Though the introduction of modern safer underground coal mining methods and automation, mine accidents still cause loss of lives, time, and money. This paper aims to analyze in detail the causes of safety and environmental issues in the coal mining industry, as well as the impact of IoT on coal mining.

**Method:** A systematic review was conducted. A comprehensive search involving Web of Science, Google Scholar, Scopus, and Science direct databases was conducted using a combination of the following keywords: mining accidents, coal mining injuries, human error in mining, intelligent mining, etc. The inclusion criteria: (1) the study was published between January 2000 and June 2020; (2) the participants were coal mining employees/coal mining accidents and accidents were work-related; (3) the study focused on identifying causes of coal mining safety issues or accidents, factors that influence unsafe behaviors and accidents in coal mining, coal mining rescue management, coal mining rescue plan, coal mining environmental impact, mining information technology, intelligent mining; (4) the study was published in a refereed journal; (5) the study was written in English. In this paper, articles were retained if they were original studies.

**Results:** A total of 59 papers were reviewed in detail. Safety issues in coal mining and the impact of IoT were identified and categorized into three main factors: general safety issues, environmental factors, and mining information technology. Recently, the coal mines had become mechanized and automated leading to improved safety, productivity, and cost. However, Human factors such as lack of appropriate skill, lack of experience, perceptual error, and unsafe behaviors, as well as lack of detailed emergency rescue plan were the leading causes of coal mining injuries. Furthermore, abandoned mining sites' carbon emission is greater than active sites.

**Conclusion:** The study recommends further research to be conducted using different psychological models to understand human factors and design effective safety management systems. And the environmental impact of abandoned mining sites should be given due attention.

## Introduction

Coal mining is one of the most dangerous industries in the world. Human factors such as intentional violation, mismanagement, and defective design cause coal mining accidents (1). The functional climate of the coal mining presents many hazards to personnel, including the proximity to machinery, hydraulic and electrical power, roof falls, and exposure to explosive mine gases and dust. Coalners are often required to work in this unsafe climate to physically control gear at short proximity and subsequently accomplish productive extraction (2, 3). Jiskani et al. (4) studied the physical and environmental working conditions of underground coal mines and discovered mining design was inappropriate were workers had to deal with excessive job demands, which lead them to face frequent ergonomic problems such as the low back, upper back, shoulder, knee, and ankle/foot pains. These injuries were associated with lack of routine, being new at the mine, and specific mining activities. A global shift toward using provisional contract labor and extended workdays indicates that injuries during long working hours will likely continue to grow as a problem in the mining industry (5). Kyeremateng-Amoah and Clarke (6) evaluated the causes of injuries in the Ghanian mining industry, where the collapse of the mine pits and falls constituted the common cause of accidents. And the injuries reported were Fractures and contusions. Similarly, underground mining reported the highest injury rate in Zambia, where the common source of fatal injuries was rockfall (7). On other hand, the occupational hazards identified in coal mining were failure to assess the work environment, failure in developing and implementing safe operating procedures, failure of workers to follow safety procedures, inadequate planning for safety in the design and operation of new equipment, and facilities (8). Even though there were great improvements both in coal mining technology and coal mine accidents, fatal injury rates in mining stay higher, where fires and explosions were the leading causes of workplace fatal injuries (9). Only technology development alone cannot achieve the broad objectives of coal mining safety, unless coupled with the wide application of risk assessment to improve coal mine safety in the future (10). Modern technologies play a vital role in this regard, for example, the application of Internet of things (IoT) in the underground mine can result in precise environment perception and early warning for flooding, fires, gas explosions, dust explosions, cave, coal and gas outburst, toxic gases, and other various risk factors. Furthermore, attain reduction of mining surface staff, hidden dangers investigation, safety hedge, accident investigation, accident emergency, miners and equipment management (11). The outcomes gained through past and present technological developments provide critical insights and lessons to help understand the value of emerging automation technologies toward achieving the future integrated mining ecosystem (3). However, human factors, lack of employees' compliance with relevant laws and regulations, decision error, perceptual error, and skill error are posing challenges to safe mining (12).

Nowadays, technological advancement and the development of new methods have been decreasing the degree of coal mining injuries and environmental impact. However, there are no sufficient review studies that present a comprehensive review of safety issues, environmental impact, and the impact of technology in reducing coal mining injures and environmental impact. Thus, knowing the safety and environmental concerns in coal mining helps to develop various types of methods for improving safety, equipment design, work procedures, work schedules, safety programs, and emergency response plans. And techniques for diagnosing the potential hazards associated with new technologies and work procedures.

## Materials and Methods

### Objective

The objective of this study was to collect papers related to coal mining safety issues, environmental impact, and the impact of IoT in coal mining, to examine and review the variables related to causes of accidents, and environmental issues, to evaluate the impact of IoT in improving coal mining safety and to present a comprehensive systematic review.

### Research Questions

The key research question of interest is the following:

(1) What are the potential causes of coal mining injuries.
(2) What are the potential environmental impacts of coal mining?
(3) What are the potential impacts of the application of information technology on reducing coal mining injuries and environmental impacts?

### Eligibility Criteria

An in-depth systematic review was conducted based on the Preferred Reporting Items for Systematic Reviews and Meta-Analyses (PRISMA) guidelines. Studies were selected based on the following inclusion criteria: (a) the study was published between January 2000 and June 2021; (b) the participants were coal mining employees/coal mining accidents and accidents were work-related; (c) the study focused on identifying causes of coal mining safety issues or accidents, factors that influence unsafe behaviors and accidents in coal mining, coal mining rescue management, coal mining rescue plan, coal mining environmental impact, mining information technology, intelligent mining; (d) the study was published in a refereed journal; (e) the study was written in English (Table 1).

**Table 1:** Studies inclusion criteria.

**Description**	**Inclusion criteria**
Publication period	Between January 2000 and June 2021
Participants of the study or Data used in the study	Coal mining employees/coal mine accidents and accidents were work-related, & data on coal mining accidents
Models, methods, or technology	Developed for the coal mining industry
The emphasis of the Study	The study focused on identifying causes of coal mining safety issues or accidents, factors that influence unsafe behaviors and accidents in coal mining, coal mining rescue management, coal mining rescue plan, coal mine environmental impact, mining information technology, and Intelligent mining
Type of Journal	Studies published in a refereed journal
Language	Studies wrote in English

### Search Strategy

**Figure 1:**
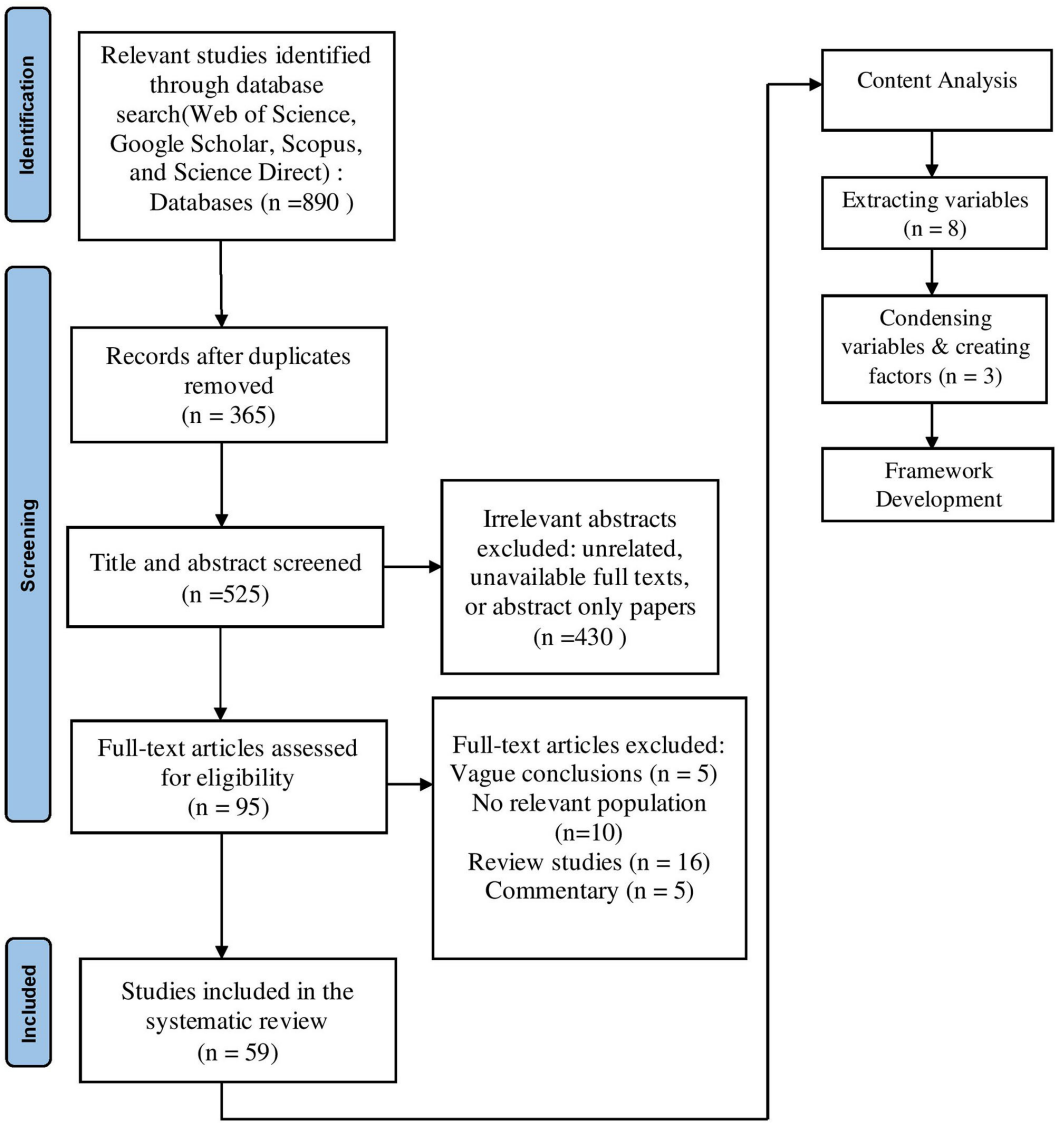
Identification and framework development.

Complete database searches were conducted between February 01 and June 30, 2021. The papers used in this review were studies of safety issues, environment, and automation of coal mining obtained via databases: Web of Science, Google Scholar, Scopus, and Science direct. The search terms were Intelligent mining, Internet of things, causes of safety issues, coal mine injuries, coal mining accidents, human error in mining, and human behavior. In total 59 articles from 2000 to 2021 were reviewed in full based on the inclusion criteria specified in Table 1. The results of the screening process are shown in the Preferred Reporting Items for Systematic Reviews and Meta-Analysis (PRISMA) diagram (Figure 1).

## Result and Discussion

### Study Selection

Figure 1 shows a flowchart of the literature search with 890 research articles identified through an initial online database search. All four authors engaged in the database screening process and the analysis of the full text for eligibility. There was no disagreement between the authors since the screening was done based on a checklist.

### Data Extraction

**Figure 2:**
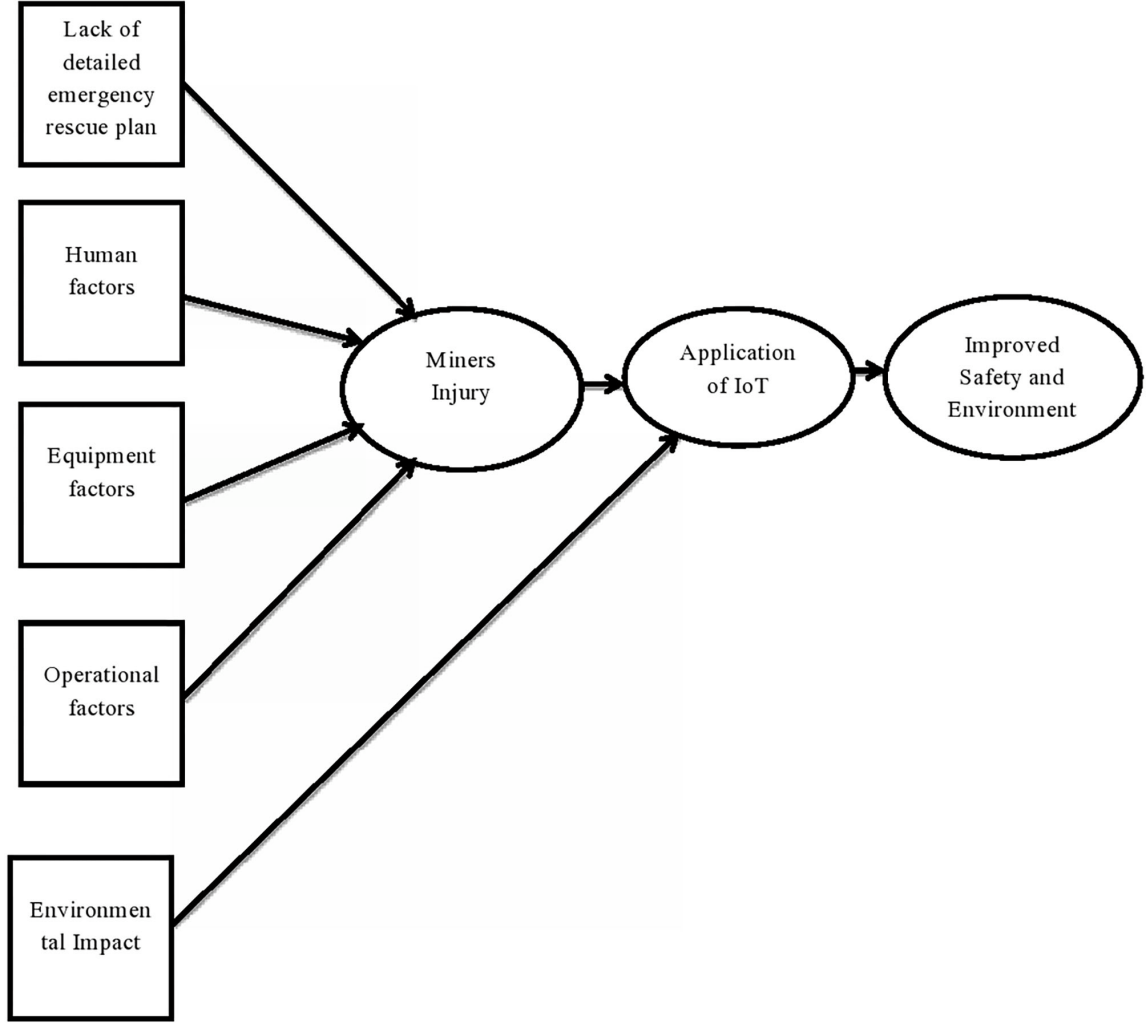
Conceptual framework of the study.

The following (Table 2) summarizes the causes of safety issues, environmental factors, and technology reported in each study: design of each study, methodology used to analyze data, the sample size, and the findings were identified in detail. The variables reported as causes of or contributory factors to safety issues and environmental factors in coal mining were analyzed based on their association with unsafe behaviors and accidents. And the impact of mining information technologies in improving safety, productivity, and cost were identified and analyzed. These variables were further condensed according to some common characteristics, which were later grouped under three categories (or factors). And the conceptual framework of the study was developed as shown below (Figure 2).

**Table 2:** Identification of causes of safety issues, environment, and the impact of technology.

**References and country**	**Study design**	**Analytical method**	**Sample size/data**	**Findings**
Chen et al. (1) (China)	Quantitative	Multi-dimensional statistical analysis	10 years data	Human factors such as intentional violation, mismanagement, and defective design were the main causes of coal mining accidents
Ralston et al. (2) (Australia)	Case study	Technology analysis		Proximity to machinery, hydraulic and electrical power, roof falls, and exposure to explosive mine gases and dust were operational hazards. Longwall automation technology improved working conditions for personnel, and enhanced environmental outcomes.
Ralston et al. (3) (Australia)	Technology analysis	Longwall mining automation analysis		Mining automation has the benefit of reduced operating cost, higher productivity, new operation culture, a reduced environmental footprint, and increased operator safety.
Jiskani et al. (4) (Pakistan)	Cross-section	Quantitative analysis	290	The frequent ergonomic problems were low back, upper back, shoulder, knee, and ankle/foot pains.
Friedman et al. (5) (US)	Secondary data analysis	Data analysis	545, 537 cases	The long working hour was the major cause of injuries.
Kyeremateng-Amoah and Clarke (6) (Ghana)	Retrospective, & cross-sectional	Descriptive data analysis	72 cases	The collapse of the mine pits and falls were the cause of accidents that result in fractures and contusions.
Michelo et al. (7) (Zambia)	Retrospective	Descriptive analysis	165 injuries & 20 fatalities	The source of fatal injuries was a rockfall
Singh and Tripathi (8) (India)	Case Study	Case analysis		Failure to assess the work environment, failure in developing and implementing safe operating procedures, failure of workers to follow safety procedures, inadequate planning for safety in the design and operation of new equipment, and facilities were hazards identified in coal mining
Smith (9) (US)	Case Study	Descriptive	172 fatal injury & 15,500 non-fatal	Fires and explosions were the leading causes of workplace fatal injuries
Chu et al. (10) (China)	Correlational study	Correlational analysis	Big data from 2001 to 2010.	The appropriate application of risk evaluation in coal mines improve coal mine safety.
Qiupinga et al. (11) (China)	IoT analysis	IoT analysis		IoT plays an vital role in decreasing mining surface personnel, hidden dangers, safety hedge, accident, accident emergency, miners, and equipment management.
Hongxia and Ruirui (12) (China)	Model analysis	HFACS and AHP analysis		Human unsafe behavior was the main cause of coal mining accidents.
Sari et al. (13) (Turkey)	Accident data analysis design	Relative frequency analysis	1,533 cases	Coal mining mechanization improves safety and productivity.
Ruff et al. (14) (US)	Big data	Data analysis	562	Operation-related injuries and machine-related accidents account for all the severe accidents in the mining industry.
Kucuker (15) (Turkey)	Retrospective data analysis	Descriptive statistical analysis	164 Fatalities	Subsidence, underground railway accidents, and methane poisoning as well as Asphyxia due to collapses was the most common causes of deaths in coal mines
Deng et al. (16) (China)	risk analysis	Coal mine risk network (CMRN) Analysis	126 accident cases	Coal mine risks were roof collapse, fire, and gas concentration exceeding the limit.
Akgun (17) (Turkey)	Case study	Case analysis	301	Impulsive combustion of coal and the presence of methane as well as weak emergency rescue systems were causes of fatalities in coal mines.
Dasha et al. (18) (India)	Data analysis	Descriptive statistics analysis	368,707 accidents	Ground movement, fall other than fall of ground, transportation machinery, machinery other than transportation machinery, and explosives were causes of accidents.
Shahani et al. (19) (Pakistan)	Fuzzy logic	Fuzzy logic data analysis		The main causes of fatalities in coal mining were mine collapse and blast, accumulation of gas, gas explosion, and falling stones
Liu et al. (20) (China)	Case study	Case analysis		Hazards in the mining process of coal: rock stresses, harmful gases, humidity, high temperatures, coal and silica dust, and specialized equipment.
Tripathy and Ala (21) (India)	Risk assessment	Risk data analysis	7,000 accidents reports	Coal mining hazards were geo-mechanical, mechanical, electrical, geochemical, and environmental.
Burgess-Limerick (22) (Australia)	Data analysis	Descriptive statistics analysis	4,633 injuries	Equipment was the major cause of injury.
Jiskani et al. (23) (Pakistan)	Survey	Descriptive statistics	330	Weak safety practices, supervisor safety, coworker safety, and job safety were causes of fatalities. Age and experience were also positively associated with mining hazards.
Ishtiaq et al. (24) (Pakistan)	Cross-sectional study	Descriptive statistics	400	Musculoskeletal, respiratory, gastrointestinal, nervous, dermatological, ear, nose & throat, and eye problems were the common problems miners face.
Carlisle and Parker (25) (Australia)	Survey	Regression analysis	231	The larger portion of the miners felt pain in at least one part of their bodies. Higher distress was also associated with greater absenteeism in workers who reported lower back pain.
Cui et al. (26) (China)	Cross-sectional	Descriptive statistics analysis	4,319	The common injuries were smashing injury, sprains, and luxation.
Chimamise et al. (27) (Zimbabwe)	Case-control study	Quantitative analysis	156 cases and 156 controls used in the study	Working underground, having targets per shift, inadequate PPE, and working more than 8 h per shift were the main source of fatalities
Sanmiquel et al. (28) (Spain)	Data mining	Scenario analysis	56,034 Mining Accidents	The immediate cause of the accident was body movement with physical effort or overexertion.
Pedram et al. (29) (Australia)	Case study	Case analysis		A competent workforce and a safe workplace were the two main components of effective management.
Palei et al. (30) (India)	Accident data analysis design	Qualitative analysis	96 Accidents and 100 Injuries	Injuries were caused due to slips, rule-based and knowledge mistakes, lapses (memory failure), and violations.
Wei-ci and Chao (31) (China)	Comparative Study design	Comparative analysis	Comparision	There is no appropriate allocation of safety and supervision budget in the Chinese coal mine. Safety training in the US coal mine is voluntary. Coal mine safety education in China is compulsory. Compared with the US, laws, and regulations of coal mines are established relatively late in China and the system is imperfect and fragmented, China has a stronger intervening impact on the production than the US.
Zhang et al. (32) (China)	Human factors analysis and classification system (HFACS) using secondary data	Descriptive statistics	94 major accidents	Causes of accidents were frequent unsafe behaviors, inadequate regulations, and failure to correct hidden danger.
Chen et al. (33) (China)	Case study	Bayesian network analysis, Fuzzy analysis	04	A worker in a poor state, commits vulnerable unsafe behaviors such as violation, and decision-making error.
Tong et al. (34) (China)	Case study	Monte carlo method	200	The risk of unsafe behaviors were ventilation, gas prevention and fire extinguishing, blasting and electrician.
Li et al. (35) (China)	Survey	Confirmatory factor analysis	593	Safety attitude was the cause of injuries
Kucuk and Ilgaz (36)	Secondary data analysis	Cause of injury analysis		These accidents were caused mainly due to technical deficiencies or failures
Duma et al. (37) (Indonesia)	Quasi-experimental design	Multi-variate data analysis	592	The work environment creates fatigue. Most injuries were caused as a result of fatigue.
Smagina et al. (38) (Russia)	Qualitative	Qualitative analysis		The human factor was the main cause of injuries.
Bhattacherjee (39) (France)	Survey	Logistic regression	516	The coal mining injuries were caused by mining equipment and human factors.
Maiti and Bhattacherjee (40) (India)	Case study	Logistic regression	6,281	Face workers were the most accident-prone job occupants than the haulage and other workers
Groves et al. (41) (Canada)	Big data	Big data analysis	190, 940 injuries data from 1995 to 2004	Non-powered hand tools were often involved with non-fatal injuries and off-road ore haulage was the source of fatalities.
Horberry et al. (42) (Australia)	Case study	Observation and case analysis	29	The key challenges for emergencies in underground coal mines were the collection and managing of information.
Cheng and Zhang (43) (China)	Qualitative	Qualitative analysis		Lack of detailed emergency rescue plan, and sound system.
Lei et al. (44) (China)	cumulative environmental effect	cumulative environmental effect analysis		The land subsidence, water resources destruction, soil erosion, air pollution, and biodiversity decrease were the environmental effect of coal mining.
Makowsky et al. (45) (China)	Case study	Laboratory analysis	14	The investigated subsided water developed for fish farming have been polluted already.
Dutta et al. (46) (India)	Laboratory test	High resolution-transmission electron microscopy (HR-TEM), energy dispersive spectroscopy (EDS), selected-area diffraction (SAED), field emission-scanning electron microscopy (FE-SEM)/EDS, X-ray diffraction (XRD), fourier transform infrared spectroscopy (FTIR), raman and ion-chromatographic analysis, and mössbauer spectroscopy.		The total sulfur content of the coal is noticeably high compared to the overburden and soil
Antoszczyszyn and Michalska (47) (Poland)	Data analysis	Data analysis		The larger portion of mercury emissions are associated with coal mining.
Rokihm et al. (48) (Indonesia)	Qualitative and quantitative	Qualitative and quantitative analysis	Data analysis	High carbon dioxide emission, deforestation, and health problems. Coal mining also harms trade balance, exchange rate, and the growth of other sectors.
Kholod et al. (49) (USA)	(MC2M) model	Scenario analysis		The emissions from the abandoned mines increase faster than those from active ones.
Ke-feia et al. (50) (Australia)	Experimental	Experimental and model analysis		The 3D modeling and collision detecting techniques simulated can provide an efficient way to represent underground situations.
Na et al. (51) (China)	Information technology analysis	Information technology analysis		Facilitate storage of the complex and bulky coal safety management information to aid the managers to arrive at the purpose of decreasing accident rates and guaranteeing safety production.
Zhi-qiang and Wei-ming (52) (China)	Analysis of quality standardization	Analysis of quality standardization		Safety quality standardization management system can enhance the mine's quality standardization success rate and reduce the frequency of the safety accidents
Runqiu et al. (53) (China)	Model analysis	Model analysis		The self-organizing data mining method improves safety
Yan et al. (54) (China)	Fuzzy mathematics	Fuzzy mathematical analysis of factors		The early warning model is effective to prevent an accident
Yinghua et al. (55) (China)	Information technology analysis	Information technology analysis		IoT technology, improved the coal mine supervising patterns.
Dong et al. (56) (China)	Information technology analysis	Information technology analysis		IoT have great significance for the safe and efficient operation of the coal equipment
Wang and Huang (57) (China)	Technological development of intelligent mining analysis	Technological development of intelligent mining analysis		Intelligent mining technology is an important advancement in coal mine safety to move miners from the dangerous work face to the safer roadway
Zhou et al. (58) (China)	Technology & risk analysis	Technology & risk analysis		Coal mine occupational safety and health management and risk control technology and the associated software can support the safety management efforts in coal mines in a standardized and effective manner
Palka and Stecuła (59) (Poland)	Technology assessment	Technology assessment analysis		Lack of technological knowledge and the incorrect training system within the company are significant problems for implementing technology in mining.

### General Safety Issues

#### Operational Factors

Coal mining safety is a very important phenomenon since coal mining involves various hazards that lead to major fatalities. In the conventional panel, the most common accident/injury types were falls, struck by a falling object, and handling material. In the mechanized panels, injuries were in haulage/transportation, machine and electricity-related (13). Machine-related accidents were conveyors, rock bolting machines, milling machines, and haulage equipment. These accidents account for all the severe accidents in the mining industry (14). Subsidence of the ground, underground railway accidents, and methane poisoning also cause fatalities (15). The operational risks were roof collapse, fire, and gas concentration exceeding the limit (16). With the spontaneous combustion of coal and the presence of methane in the structure of the lode, underground coal mining has the highest rate of fatal accidents and injury in the mining industry (17). Ground movement, fall other than fall of ground, transportation machinery, machinery other than transportation machinery, and explosives also contributed to accidents caused in the mining sites (18). For example, in Pakistan, the main causes of fatalities in coal mining were mine collapse and blast, accumulation of gas, gas explosion, and falling stones. The fatality rate was higher because of a lack of safety training and education, child labor and illegal mining, and a lack of appropriate technology (19). On the other hand, rock stresses, harmful gases, humidity, high temperatures, coal and silica dust, and specialized equipment were the potential operational hazards that lead to severe accidents (20). Furthermore, geo-mechanical, mechanical, electrical, geochemical, and environmental were potential hazards causing miners injuries (21). Shuttle car injuries most frequently occurred to drivers as a consequence of traveling over rough roads or being struck by and during maintenance (22).

Musculoskeletal disorders, work design, ground or strata instability, and biological sources were hazards associated with underground miners task (23). Individual factors like age and job experience, as well as physical and environmental job factors including inappropriate work pace, restricted working space, manual material handling tasks, poor lighting conditions, significantly, contributed to musculoskeletal injuries among miners (4). Despite improvements, fatal injury rates in mining remain more than four times higher than the average for all industries in the US, where fires and explosions were the leading causes of workplace fatal injuries (9). Coal miners were forced to work in difficult temperatures, postures, and work conditions, which leads to various occupational health problems. They reported musculoskeletal, respiratory, gastrointestinal, nervous, dermatological, ear, nose & throat, and eye problems among the frequent occupational health problems (24). Due to the nature of the coal mining operation, miners experienced pain in at least one region of their bodies (25). The larger portion of the injuries that occurred in underground mining was smashing injury, sprains, and luxation (26). According to Chimamise et al. (27), the factors associated with severe injuries in mining were working underground, having targets per shift, inadequate personal protective equipment (PPE), and working more than 8 h per shift. The immediate cause of the accident was body movement with physical effort or overexertion, and the type of accident was physical effort or overexertion (28).

#### Human Factor

In the workplace, most of the time accidents occur from human errors or uncontrolled situations. Human error is defined as an action, intentional or harming safety or productivity (29). The main injuries were slips, followed by rule-based and knowledge mistakes, lapses (memory failure), and violations (30). The frequent occurrence of mine disasters was largely attributed to poor management, weak enforcement of legislation and policies, lack of safety awareness among the mining communities, poor involvement of government, civil society organizations, and the private sectors, and insufficient safety education (31). Failure to correct hidden danger, failure to implement a policy of coal mine production, and failure to guide safety in production were the top three serious human factors/issues in coal mining (32). These unsafe behaviors were when a worker is in a poor state, the most vulnerable unsafe behaviors are a violation, followed by decision-making error. And lack of experience was the most significant contributory factor to unsafe behaviors, and poor fitness for duty was also the principal state that causes unsafe behaviors (33). For example, coal mining gas explosion accidents were caused by unsafe worker's behaviors (34). The perception of miners plays a vital role in their unsafe behavior, iners perceived lower levels of management safety practices, followed by supervisor safety, coworker safety, and job safety. Age and experience were significantly associated with mining hazards The higher prevalence of mine hazards predicts poor workplace safety and a low safety climate (23). The safety attitude of miners are crucial in identifying mine hazards and decreasing coal mine operation injuries. Safety attitude is positively related tosafety behavior.afety participation and safety compliance also positively related to safety attitude. Age and length of service were also slightly related to safety attitude (35). Firedamp and dust explosions, landslips, mine fires, and technical failures related to transport and mechanization were the common causes of coal mining accidents in Turkey. These accidents were caused mainly due to technical deficiencies or failures (36). According to Duma et al. (37) in coal mining, most of the injuries were caused a result of fatigue. The problem of production safety increase at coal mines can be solved not only by expanding technical, technological, and organizational measures but also by exploiting the human factor. Thus, to decrease injuries in coal mining, emphasis should be given to the increase in the level of communicative and psychological competence of managers and specialists, and the combination of material incentives and disincentives for compliance with safety requirements (38).

#### Mining Equipment Factor

In coal, mining accidents occur due to operational and human factors. However, the share of equipment factors also cannot be undermined. Accidents occur from the failure of a Power hammer, vibrating hand tools, pneumatic tools, and bent trunk cause severe injuries (39). In coal mining, face workers were the most accident-prone job occupants than the haulage and other workers (40). Despite significant intervention and the reduction, the number of coal mining injuries and fatalities remains high. The non-powered hand tools were the equipment category most often involved with non-fatal injuries while off-road ore haulage was the most common source of fatalities (41). In mechanized panels manual handling-related injuries were found to be higher than in conventional panels (13).

#### Lack of Emergency Rescue Plan

The emergency rescue plan was not more than a paper document that had not been properly tested, most mines had not formally identified what information would be necessary for an emergency, absence of training carried out in emergency preparedness and response especially in the management of incidents and there was no industry-wide competency standard for control room operators (42). There had been guidelines and requirements associated with coal mine emergency rescue work, however, there is no sound system, solely part of it. The vast regulations can't precisely and successfully deal with specific coal mine emergencies and lack coal mine emergency rescue regulations and requirements (43). The emergency management capability and rescue capacity were still insufficient. Limited investment of many enterprises, even though the law requires each enterprise to have its plan of emergency preparedness; however, companies only have general plans which are far behind the actual situations, the emergency management techniques of shallow mining cannot secure the safe production of coal mines. Weak emergency rescue systems also greatly contribute to fatalities in coal mines. The occurrence of these accidents and casualties despite technological advances indicates that adequate precautions had not been taken (17).

### Environmental Impact of Coal Mining

The underground mining extracting coal resources through the wells typically leads to land subsidence, water resources destruction, soil erosion, air pollution, and biodiversity decrease. These issues can have interactions with each other, and develop through time and space, which speed up the environmental deterioration of the coal mining area (44). The investigation of subsided water areas in the Panji coal mining area evidenced twelve subsided water areas developed for fish farming have been polluted already (45). On other hand, the assessment of Indian coal mining acid drainage revealed the total sulfur content of the coal is noticeably high compared to the overburden and soil. The coal mine water was also highly acidic in nature (46). The content of mercury in soils in areas degraded by mining and processing of coal is very high, compared to the geochemical background (47). Coal mining is the largest contributor to the global carbon dioxide emission, deforestation of dense forests, and exposes communities living around the mine site to severe health problems (48). The consequence of methane emission to the atmosphere from mining sites doesn't end with the abandonment of the mine site. The emission from abandoned coal mining sites increases rapidly than those of active sites (49).

### Mining Information Technology

The introduction of technological changes in mining operations speed up enhancement in productivity, health, and safety. Intelligent response and rescue systems, have contributed drastically to the reduction of mining fatalities and accidents. The underground positioning technique and the 3D modeling and representing technique developed can satisfy the requirements of underground rescue in an emergency (50). The safety information management system based on the internet, web, common gateway interface, active server page, and hypertext preprocessor technologies are widely admired by many coal enterprises. This was due to the fact that they facilitate storage of the complicated and bulky coal safety management information in classifications, allowing the safety management to be more timely, more efficient, and more accurate, to aid the managers to arrive at the purpose of reducing accident rate and guaranteeing safety production (51). Using fuzzy assessment technique managers and personnel can rapidly determine the mine's current safety quality standardization level, to grate, discover, and dispose of the hidden mine safety problems in time, which enhance the efficiency of security management. Coal mine safety quality standardization management system has the benefit of the low cost of development, ease of maintenance, and pleasant interface (52).

Runqiu et al. (53), introduced a self-organizing data mining technique that spontaneously analyzes non-linear relation between the gas emission and the factors, and can establish the explicit high order equation to descript the gas emission laws and the prediction model which has enough prediction accuracy for the application of actual engineering in coal mines. The safety early warning model of coal mining is effective to prevent mining accidents (54).

The application of IoT in the underground coal mining can attain unique environment perception and early warning for flooding, fires, gas explosions, dust explosions, cave, coal and gas outburst, toxic gases, and other various risk factors. Further, reduce mining surface personnel, hidden dangers investigation, safety hedge, accident investigation, accident emergency, miners and equipment management (11). The remote dynamic supervision can be innovated through adopting IoT technology. The tracking inspection on illegal action can be achieved, capabilities of emergency response and accident investigation can be increased, the situation of safe production can be further improved, and safe and stable development of coal industry can be promoted (55). It provides a new trend of thought for the development of coal informatization. Since the coal extraction process is done in shifts, the coal mining equipment was run continuously for a long period under difficult site conditions leading to a series of problems. Traditional maintenance mode is difficult to find the failure accurately and timely in the early days. IoT technology can identify the fault and forecast the potential threat accurately, and it is of great significance for the safe and efficient operation of coal equipment (56).

The introduction of Intelligent mining technologies has reduced significantly the number of miners on the work face. Miners only monitor mining machines on the roadway or or at the surface control center since intelligent mining can be applied to extract middle-thick or thick coal seams. As a result, miners' safety has been improved (57). The introduction of coal mine occupational safety and health management and risk control technology system and its supporting software, successfully drive coal mine work-related safety and health management, while decreasing the accident risks to provide safety assurance to coal mining operations. Further, the system can be carried out based on a benign cycle with dynamic feedback and scientific development (58).

The outcomes gained via previous and present technological advancement deliver critical insights and lessons to help understand the value of emerging automation technologies toward achieving the future integrated mining ecosystem (3). However, for the implementation of suitable technology, the selection of the right personnel is a growing problem in mining. This was because of a lack of technological knowledge and the improper training system within the company. The implementation of cyber-physical systems can deepen the frustrations of employees. Thus, a well-educated, prepared, motivated, and, above all, aware and committed employee is the basis for the development and success of every company (59).

### Limitations

The study doesn't conduct a statistical meta-analysis study due to the variety of methodologies that have been applied in the studies, and this could have produced a more reliable conclusion. Furthermore, we have done an extensive literature search from four online databases and used inclusive search terms, we were unable to rule out the likelihood of failing to spot some relevant articles.

## Conclusions

Despite improvements in safety performance, the number and severity of mining-related injuries remain high and unacceptable, indicating that further reduction can be achieved. Safety issues in coal mining were operational factors, human factors, equipment factors, environmental factors, and lack of detailed emergency rescue plans. The finding of the study shows, in recent years most of the coal mines have become mechanized and automated leading to a decreased number of fatal injuries and improved productivity. However, human factors: lack of skilled manpower that can easily adapt to new coal mining technologies, lack of experience, perceptual error, and unsafe behaviors continue to pose a great challenge to the future of the industry. The lack of a detailed emergency rescue plan with each mining site's physical conditions was also an important source of injuries in the mining sector. Furthermore, studies confirm that the environmental impact of mining sites even becomes greater in terms of carbon emission beyond the abandonment of the coal mining sites. More emphasis should be given to abandoned mining sites to reduce their environmental impact.The limitations of the studies used in this systematic review were lack of clear methodology in some studies, the small sample size was also used in some studies, most of the studies used secondary data from newspapers and hospitals, which is affected by under-reporting, and over-reporting. Furthermore, the studies had not reported the importance of near-miss injuries reports in designing effective coal mine safety. Thus, further research should be conducted using different psychological models to understand the causes of unsafe human acts and design effective safety management systems.

## Data Availability Statement

The original contributions presented in the study are included in the article/supplementary material, further inquiries can be directed to the corresponding author/s.

## Author Contributions

GB designed and conceptualized the study and wrote the manuscript. LY supervised the project and obtained funding. JG and JZ download the related papers and also obtained funding. All authors participated in screening the articles and provide critical feedback, significantly contributed to the study, and approved the final manuscript.

## Conflict of Interest

The authors declare that the research was conducted in the absence of any commercial or financial relationships that could be construed as a potential conflict of interest.

## Publisher's Note

All claims expressed in this article are solely those of the authors and do not necessarily represent those of their affiliated organizations, or those of the publisher, the editors and the reviewers. Any product that may be evaluated in this article, or claim that may be made by its manufacturer, is not guaranteed or endorsed by the publisher.
